# 3*β*-Acetyloxy-oleanolic Acid Attenuates Pristane-Induced Lupus Nephritis by Regulating Th17 Differentiation

**DOI:** 10.1155/2019/2431617

**Published:** 2019-05-22

**Authors:** Xiaoqing Zhou, Huanpeng Chen, Fengjiao Wei, Qingyu Zhao, Qiao Su, Jinhao Liang, Meng Yin, Xuyan Tian, Zhonghua Liu, Bolan Yu, Chuan Bai, Xixin He, Zhaofeng Huang

**Affiliations:** ^1^Institute of Human Virology, Sun Yat-Sen University, Guangzhou, China; ^2^Department of Biochemistry and Molecular Biology, Zhongshan School of Medicine, Sun Yat-Sen University, Guangzhou, China; ^3^Sun Yat-Sen University Cancer Center, Guangzhou, China; ^4^Animal Experiment Center, The First Affiliated Hospital of Sun Yat-Sen University, Guangzhou, China; ^5^School of Pharmaceutical Sciences, Guangzhou University of Chinese Medicine, Guangzhou, China; ^6^Animal Experiment Center, South China Agricultural University, Guangzhou, China; ^7^Key Laboratory for Major Obstetric Diseases of Guangdong Province, Third Affiliated Hospital of Guangzhou Medical University, Guangzhou, China

## Abstract

Th17 activity has been implicated in systemic lupus erythematosus (SLE), which is a systemic autoimmune disease with a typical clinical manifestation of lupus nephritis (LN). Retinoic acid receptor-related orphan receptor gamma t (ROR*γ*t) has been shown to be important for Th17 differentiation. In this study, we evaluated the inhibition of ROR*γ*t activity by 3*β*-acetyloxy-oleanolic acid (AOA), a small molecule isolated from the root of *Symplocos laurina*, a traditional herb belonging to South China. We demonstrated that AOA can inhibit ROR*γ*t activity and prevent SLE pathogenesis in a pristane-induced LN model. The results showed that AOA decreased ROR*γ*t transcription activity in a reporter assay and prevented Th17 differentiation *in vitro*. *In vivo* studies showed that AOA treatment decreased serum anti-dsDNA antibody and alleviated renal pathologic damage as well as antibody complex accumulation in the pristane-induced LN model. These results demonstrated that AOA can improve the clinical manifestation of LN, indicating potential application in SLE therapy.

## 1. Introduction

Systemic lupus erythematosus (SLE) is an autoimmune disease characterized by systemic inflammation, multiple organ injury, and the production of multiple autoantibodies [1, 2]. The pathogenesis of SLE is complex and influenced by multiple factors, including genetics, environmental factors, immune abnormalities, and epigenetics. Lupus nephritis (LN) is a typical clinical manifestation of systemic lupus erythematosus (SLE) [3]. Numerous studies have demonstrated that Th17 cells play a fundamental role in mediating autoimmune disorders, such as SLE, experimental autoimmune encephalomyelitis (EAE), and collagen-induced arthritis (CIA) [4–6]. Th17 cells produce key cytokines, including IL-17A, IL-17F, and IL-23 [7]. Loss of function of IL-17A and IL-17F can significantly reduce mortality rates and decrease renal injury in lupus nephritis mouse models [8, 9]. Similarly, IL-23R deficiency can alleviate renal damage in lupus-prone animals [10]. These studies demonstrated that Th17 cells can regulate SLE pathogenesis via different characteristic cytokines.

ROR*γ*t is a key transcription factor for the development of Th17 cells and IL-17 secretion [11, 12]. Deficiency of ROR*γ*t alleviated the manifestation of multiple autoimmune disorders, such as experimental allergic encephalomyelitis (EAE), SLE, and rheumatoid arthritis (RA) [13–15]. Some studies have reported that small-molecule inhibitors prevent the development of autoimmune disease by inhibiting ROR*γ*t activity. Digoxin can inhibit ROR*γ*t activity and decrease the clinical score and mortality rate of EAE [16]. Ursolic acid (UA), isolated from many fruits, can also prevent the development of EAE by blocking Th17 differentiation [17]. Although many ROR*γ*t inhibitors have been reported, therapeutic targeting of ROR*γ*t is still in infancy, as only a small number of molecules have advanced to clinical testing.

*Symplocos laurina* Wall belongs to *Symplocos srtchuensis Brand* (*symplocaceae*), which is indigenous to southern China [18]. Many species of this genus have been used as traditional herbal medicines for nephritis [19, 20]. The genus *Symplocos* mainly contains chemicals shown to have diverse biological activities, particularly anti-HIV activity, antitumor applications, antibacterial effects, and inhibitory activities against phosphodiesterase [21]. The compound 3*β*-acetyloxy-oleanolic acid (AOA) was isolated from the root of *Symplocos laurina* Wall [22]. However, its biologic activity remains unclear.

In this study, we assessed the potential anti-inflammatory activity and therapeutic effects of AOA in LN and its therapeutic role in the treatment of Th17-mediated autoimmune diseases.

## 2. Materials and Methods

### 2.1. Ethics Statement

All of the animal experiments were approved by the Ethics Committee of ZSSOM on Laboratory Animal Care (No. 2017-273) and were performed according to the guidelines of the Institute for Laboratory Animal Research of Sun Yat-sen University Laboratory Animal Center (Guangzhou, China).

### 2.2. Mice

We used 6-8-week-old C57BL/6J female mice for T cell differentiation *in vitro* experiments. We used 8-10-week-old BALB/c female mice to establish a nephritis model. All of the animals were purchased from the National Resource Center for Mutant Mice of China (Nanjing, China). All of the mice were housed under specific pathogen-free conditions with a 12-h light/dark cycle at 22°C in Sun Yat-sen University Laboratory Animal Center (Guangzhou, China).

### 2.3. BALB/c Mouse Models of Pristane-Induced Lupus Nephritis

BALB/c female mice at 2 months old received a single intraperitoneal injection of 500 *μ*L of pristane (Sigma Aldrich, MO, USA) [23]. Mice that were injected with 500 *μ*L of saline served as normal controls (*n* = 6). Pristane-induced LN mice were randomized into the following three groups: (1) AOA-treated group (50 mg/kg dissolved in 25% ethanol and 75% hydroxypropyl betadex, *n* = 12); (2) prednisone acetate-treated group as the positive control (15 mg/kg dissolved in 25% ethanol and 75% hydroxypropyl betadex, *n* = 16), prednisone acetate tablets were purchased from Guangdong Huanan Pharmaceutical (Guangzhou, Guangdong, China); and (3) model group (25% ethanol and 75% hydroxypropyl betadex, *n* = 16). Treatments were administered by oral gavage twice weekly for 2 months.

### 2.4. Preparation of AOA

#### 2.4.1. Plant Material

The root of *Symplocos laurina* Wall was collected from Gangkou Town, Huizhou City, Guangdong Province, China, in October 2012. Dr. Guangtian Peng was responsible for the identification of the plant. A voucher specimen (No. HXX-001) was deposited in the Department of Materia Medical Chemistry, Guangzhou University of Chinese Medicine.

#### 2.4.2. Extraction and Isolation

The AOA was prepared following our previous work. The air-dried root of *Symplocos laurina* Wall (30 kg) was powdered and extracted with 95% ethanol at room temperature for 24 h in 4 cycles. After removal of the solvent under reduced pressure, the brown extract (860 g) was suspended with water and sequentially partitioned with ethyl acetate and n-butanol. The acetyl acetate extract (500 g) was subjected to column chromatography (CC) on silica gel (200-300 mesh) with increasing concentrations of acetyl acetate in petroleum ether. The fraction (petroleum ether-ethyl acetate 9/1, *v*/*v*) was collected and resubjected to CC on silica gel to yield AOA (850 mg, C_32_H_50_O_4_, MW, 498.74, purity of 98.1%), which was determined by comparison to published NMR data (Figure S1) [22].

### 2.5. Cell Culture

ROR*γ*t-Jurkat reporter cell lines were established using previously published instructions [24].

#### 2.5.1. Luciferase Reporter Assays: EC50 Assay

ROR*γ*t-Jurkat cells (4 × 10^5^/mL) were seeded into 96-well round-bottom plates and cultured with the compound AOA (0.08, 0.4, 2, and 10 *μ*M). Cells were lysed 6 h later, and the half-maximal effective concentrations (EC50) were determined.

#### 2.5.2. Cell Viability Assays: CC50 Assay

ROR*γ*t-Jurkat cells (2 × 10^5^/mL) were seeded into 96-well round-bottom plates and cultured with the compound AOA (0.08, 0.4, 2, and 10 *μ*M). After 48 h, MTT (dimethylthiazolyl-2-5-diphenyltetrazoliumbromide) was added and incubated at 37°C for 4 h, and then, the supernatant was discarded. The optical density (OD) was then measured at 495 nm, and the value of CC50 was calculated.

#### 2.5.3. T Cell Differentiation *In Vitro*

CD4^+^CD25^−^ T cells were purified using a MACS magnetic column with a CD4^+^ T cell negative enrichment kit according to the manufacturer's protocol (eBioscience, USA). Native CD4 T cell was activated with anti-CD3e antibody (5 *μ*g/mL, eBioscience) and anti-CD28 antibody (2 *μ*g/mL, eBioscience) in 12-well plates. Cultures were supplemented with mouse IL-6 (30 ng/mL, R&D Systems, Minneapolis, MN, USA), human TGF-*β* (5 ng/mL, R&D Systems), mouse IL-1*β* (20 ng/mL, R&D Systems), anti-mouse IL-4 antibody (5 *μ*g/mL, eBioscience), and anti-mouse IFN-*γ* antibody (5 *μ*g/mL, eBioscience).

### 2.6. Enzyme-Linked Immunosorbent Assay (ELISA) for Serum Anti-dsDNA Antibody Measurement

Anti-dsDNA antibody was measured by ELISA using an in-house ELISA kit. The protocol for the detection of anti-dsDNA antibody has been described previously [25].

### 2.7. Renal Histology and Immunoglobulin Deposition

#### 2.7.1. Renal Histopathologic Analysis

Kidneys were soaked in 4% polyoxymethylene for 24 h, embedded in paraffin, and then sectioned at a thickness of 4-6 *μ*m. Sections were stained with Periodic acid-Schiff (PAS). We evaluated the severity of renal impairment using a semiquantitative scoring system (0: no involvement, 1: mild involvement of 0–30%, 2: moderate involvement of 31–60%, and 3: severe involvement of >60%) to assess 6 different parameters (glomerular volume, mesangial hypercellularity, endocapillary cellular infiltrate, endocapillary cellular crescents, interstitial crescents, and interstitial inflammatory infiltration). The glomerular indices were determined by examining 10 to 15 glomeruli to determine the average score.

#### 2.7.2. Immunofluorescence Detection

Kidney sections were stained with Alexa Fluor 488 goat anti-mouse IgG (H+L) (Invitrogen, Carlsbad, CA, USA) and Alexa Fluor 488 goat anti-mouse IgM (*μ* chain) (Invitrogen, Carlsbad, CA, USA). The antibody was diluted at 1 : 200. The IgG and IgM depositions in glomerulus were evaluated by measurement of fluorescence intensity in a total of 10-15 randomly selected glomerulus per section and scored blindly on a scale of 0–3 (0: none, 1: weak, 2: moderate, and 3: strong).

### 2.8. Flow Cytometry

Intracellular cytokine staining was performed according to the manufacturer's protocol of the Mouse Foxp3 Buffer Set (BD Biosciences) and analyzed using FlowJo software. The following reagents were used: Pacific Blue anti-mouse CD4 (eBioscience), PE anti-mouse TCR-*β* (BD Biosciences), PE anti-mouse ROR*γ*t (eBioscience), FITC anti-mouse IFN-*γ* (eBioscience), and APC anti-mouse IL-17A (eBioscience).

### 2.9. RNA Isolation and Quantitative RT-PCR

Total RNA from splenocytes were extracted using TRIzol (Invitrogen). RNA (1 *μ*g) was reverse-transcribed into cDNA using the PrimeScript RT Reagent Kit (Takara Bio, Kusatsu, Japan). Gene expression was determined using quantitative real-time PCR (Takara Bio, Kusatsu, Japan). The relative expression was calculated by normalizing the expression of each target to glyceraldehyde-3-phosphate dehydrogenase (GAPDH) using the 2-∆∆Ct method. Quantitative RT-PCR was performed using the primers listed in Table S1.

### 2.10. Statistical Analysis

Data are expressed as mean ± SEM. The statistical significance between groups was determined by one-way analysis of variance followed by Bonferroni's test and Student's *t*-test. *p* < 0.05 was considered to be statistically significant. Statistical analyses were performed using GraphPad Prism 5.0 (GraphPad Software Inc., San Diego, CA, USA).

## 3. Results

### 3.1. AOA Inhibited ROR*γ*t Transcription Activity

**Figure 1:**
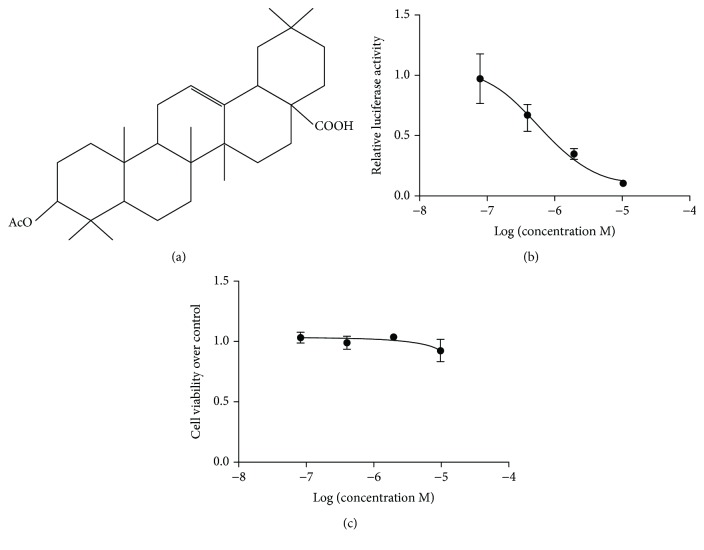
The effect of AOA on the ROR*γ*t-Jurkat cell line. (a) The structure of AOA (3*β*-acetyloxy-oleanoic acid). (b) The effect of AOA (0.08, 0.4, 2, and 10 *μ*M) on the ROR*γ*t-Jurkat cell line by luciferase reporter activity assays—EC50 assays. (c) The effect of AOA (0.08, 0.4, 2, and 10 *μ*M) on the ROR*γ*t-Jurkat cell line by MTT assays—CC50 assays. Data were representative of three independent experiments.

AOA, which is used in traditional herbal medicine in South China, was isolated from the root of *Symplocos laurina* Wall. The structure of AOA is shown in Figure 1(a). In previous studies, we established a stable ROR*γ*t-Jurkat cell line to test the activity of ROR*γ*t antagonists [24]. In this study, AOA exhibited potent inhibitory effects on ROR*γ*t transcription activity, with an EC50 value of 0.9483 *μ*M (Figure 1(b)). The cytotoxic effect of AOA was analyzed by MTT assays, and the results showed that ROR*γ*t-Jurkat cells were only poorly sensitive to AOA with a CC50 value of 23.96 *μ*M (Figure 1(c)). The ratio of CC/EC > 20, demonstrating the potency of AOA for drug development.

### 3.2. AOA-Mediated Dose-Dependent Inhibition of Th17 Differentiation

**Figure 2:**
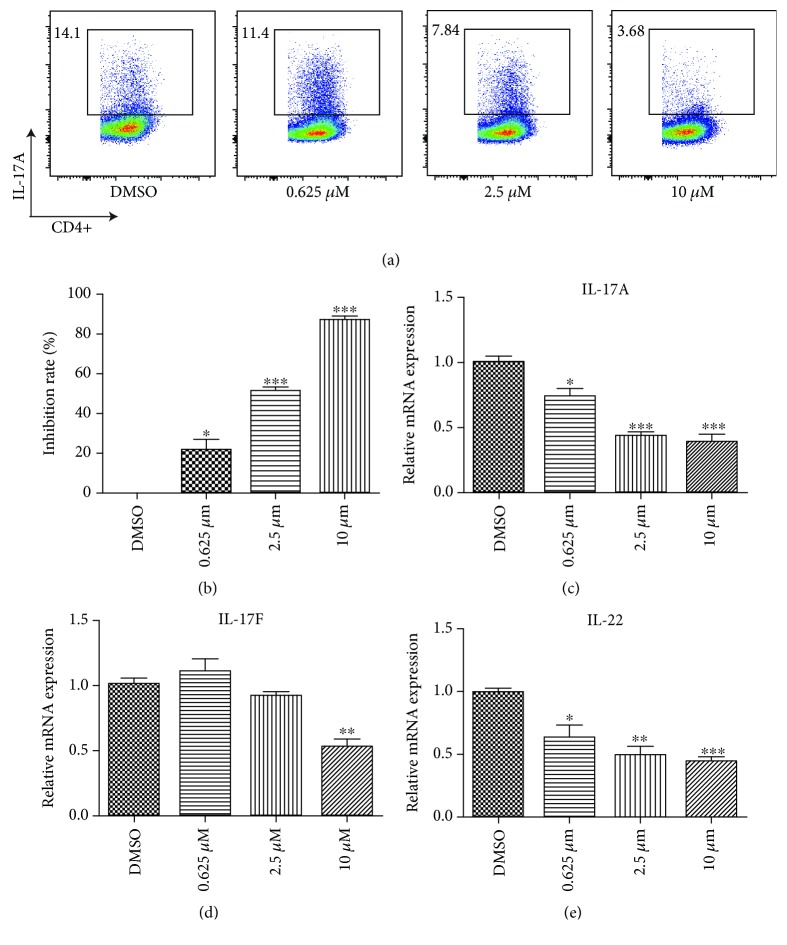
AOA dose-dependent inhibition of Th17 differentiation. Native CD4^+^CD25^−^ T cells were subjected to Th17 cell differentiation protocols as described in Materials and Methods. (a) Flow cytometry analyzing intracellular IL-17A in native CD4^+^CD25^−^ T cells with AOA (0.625, 2.5, and 10 *μ*M) under mouse Th17 cell differentiation conditions. (b) The rate of inhibition was calculated vs. DMSO group. (c–e) *IL-17A*, *IL-17F*, and *IL-22* expression was quantified and normalized to GAPDH. The *in vitro* differentiation and quantitative real-time PCR were repeated 3 times with consistent results. The results are shown as mean ± SEM. ^∗^ *p* < 0.05, ^∗∗^ *p* < 0.01, and ^∗∗∗^ *p* < 0.001.

Since ROR*γ*t is required for Th17 cell differentiation, we next investigated whether AOA could sufficiently inhibit Th17 cell differentiation. We performed *in vitro* Th17 cell differentiation in the presence of different concentrations of AOA. We found that AOA inhibited mouse Th17 cell differentiation in a dose-dependent manner (Figures 2(a) and 2(b)). As expected, AOA significantly inhibited the transcriptional expression of *RORγt*. Additionally, the mRNA levels of the inflammatory cytokines *IL-17A*, *IL-17F*, and *IL-22* were significantly decreased, with increasing concentrations of AOA (Figures 2(c)–2(e)). However, AOA had little effect on the expression of Th1 and Th2-related cytokines and transcription factors. The mRNA levels of the IFN-*γ*, Tbx-21, IL-4, and IL-13 were not significantly changed with increasing concentrations of AOA. The expression of Foxp3 increased when AOA concentration was 10 *μ*M, suggesting that high AOA concentration promoted the function of Treg cells in some extent (Figure S2).

### 3.3. AOA Significantly Reduced the Serum dsDNA Level in a Mouse Model of Lupus Nephritis

**Figure 3:**
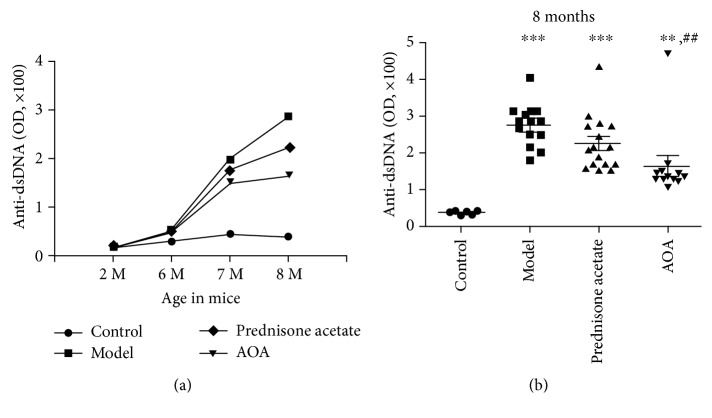
AOA can significantly reduce serum dsDNA levels in a mouse model of lupus nephritis. (a, b) Serum from the mice in each group was collected at 2 months old (pristine injection), 6 months old (4 months after pristine injection), 7 months (one month after AOA treatment), and 8 months (two months after AOA treatment—the endpoint of this experiment), and the antibody levels were detected by ELISA. Normal controls (*n* = 6), model controls (*n* = 14), positive drug group-prednisone acetate (*n* = 15), and AOA-treated group (*n* = 12). The data were repeated 3 times with consistent results. Data are presented as mean ± SEM. ^∗^ *p* < 0.05, ^∗∗^ *p* < 0.01, and ^∗∗∗^ *p* < 0.001, calculated versus the control group; ^#^ *p* < 0.05 and ^##^ *p* < 0.01 calculated versus the model group.

To address the therapeutic potential of AOA in Th17-mediated autoimmune diseases, we tested the effect of AOA on pristane-induced lupus mice. In this study, pristane was injected into 2-month-old mice. At 6 months of age (4 months after the pristine injection), these mice received AOA twice per week for two months. Animals were killed at 8 months old for gross pathological observation of LN. A notable feature of LN is the production of dsDNA antibodies associated with renal damage. Serum dsDNA was detected at the following time points: 2 months of age (before pristane treatment), 6 months old (4 months after pristane treatment), 7 months (one month after AOA treatment), and 8 months (two months after AOA treatment—experimental endpoint). Serum was collected to detect the level of anti-dsDNA antibodies. There were marked increases in serum anti-dsDNA antibody levels in the model group at months 7 and 8 compared with the control group. However, the anti-dsDNA antibody titer was markedly decreased in the AOA-treated group in comparison to the model group (Figures 3(a) and 3(b)).

### 3.4. AOA Reduced the Production of Inflammatory Cytokines in Mice with Lupus Nephritis

**Figure 4:**
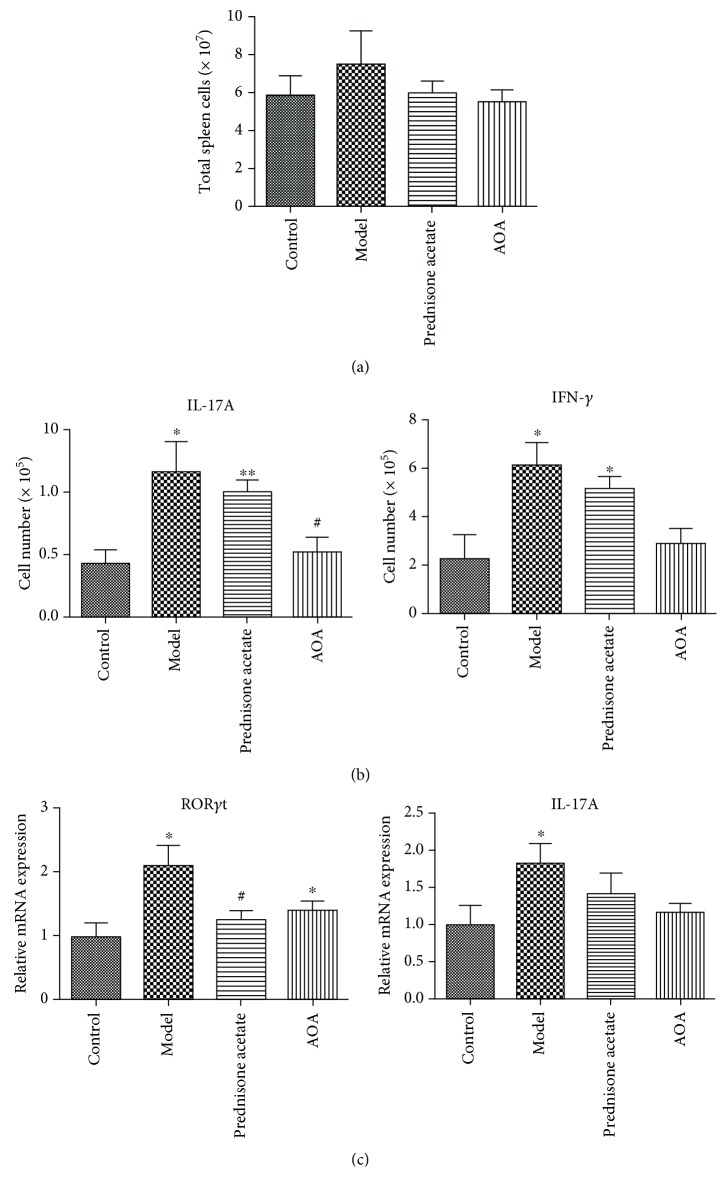
AOA can significantly reduce the production of inflammatory cytokines in a mouse model of lupus nephritis. Spleen lymphocytes were collected from normal controls, model control group, prednisone acetate-treated group (15 mg/kg), and AOA-treated group (50 mg/kg) at 8 months old. (a) The total number of spleens. (b) Flow cytometry analyzing the total number of IL-17A+ and IFN-*γ* ^+^ cells. (c) The mRNA expression levels of *RORγt* and *IL-17A* in the spleen of lupus nephritis mice. The quantitative real-time PCR were repeated 3 times with consistent results. Data are presented as mean ± SEM. ^∗^ *p* < 0.05 and ^∗∗^ *p* < 0.01, calculated versus the control group; ^#^ *p* < 0.05, calculated versus the model group.

Flow cytometry was used to assess the expression of IL-17A and IFN-*γ*. There was no significant difference in the total number of spleens; however, the AOA-treated mice contained fewer IL-17A+ cells and IFN-*γ* ^+^ cells in the spleens (Figures 4(a) and 4(b)).

Quantitative PCR (qPCR) analyses of splenic cells revealed decreased mRNA levels of *RORγt* and *IL-17* in AOA-treated mice compared with model controls (Figure 4(c)).

### 3.5. AOA Alleviated Renal Damage in Mice with Lupus Nephritis

**Figure 5:**
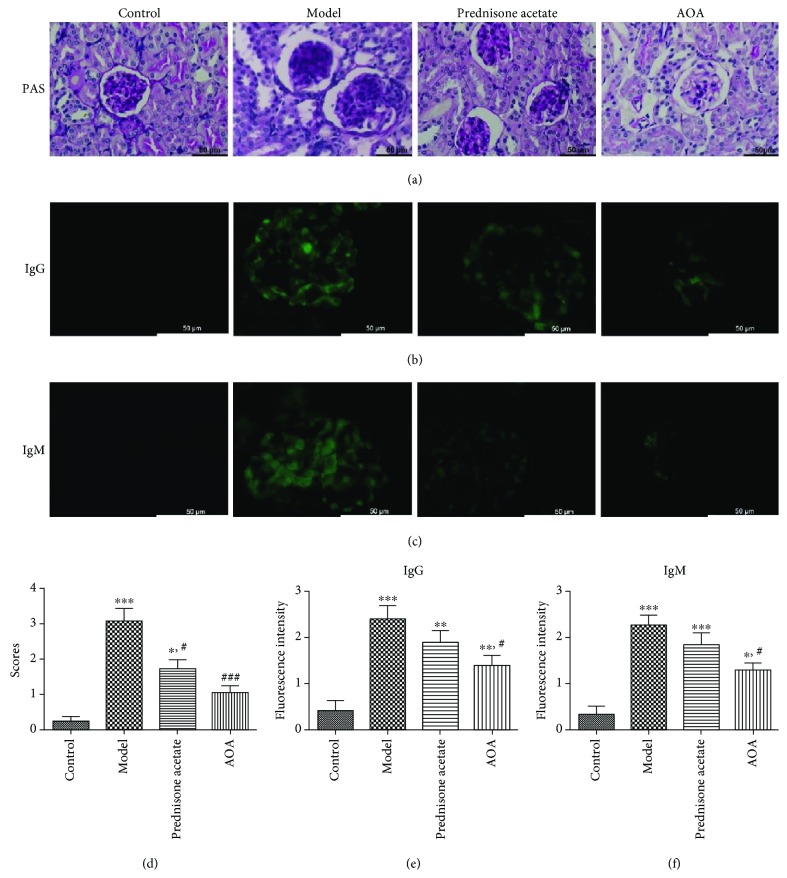
AOA can alleviate renal damage in a mouse model of lupus nephritis. Representative photomicrographs of kidneys from controls and lupus nephritis mice at 8 months are shown. (a) PAS staining of healthy kidney tissues and tissues from lupus nephritis mice. (b, c) Immunofluorescence analysis with an anti-IgG and anti-IgM antibody for detecting immunoglobulin deposition. (d) Histopathologic scores for four different groups are shown according to PAS staining results. (e, f) Fluorescence intensity of IgG and IgM depositions is shown. 10-15 glomeruli were examined, and an average score was obtained. Data are presented as mean ± SEM. ^∗^ *p* < 0.05, ^∗∗^ *p* < 0.01, and ^∗∗∗^ *p* < 0.001, calculated versus control group; ^#^ *p* < 0.05, ^##^ *p* < 0.01, and ^###^ *p* < 0.001, calculated versus model group.

Histomorphology examination of kidneys with PAS staining and immunofluorescence (IF) analysis with an anti-IgG and anti-IgM antibody were performed to detect immune complex deposition and renal damage. Consistent with the foregoing observations, the model control group mice showed a remarkable change in glomerular histology, including enlarged of glomerular volume, increased mesangial expansion, basement membrane thickening, and lymphocytic infiltration. However, the AOA-treated group exhibited reduced glomerular damage compared with the model control group mice with lupus (Figure 5(a)). Histopathology scores were based on PAS staining, and were significantly higher in the model group of mice than in the control mice; however, an obvious decline was observed in the AOA-treated group (Figure 5(d)). In the experiment investigating immune complex deposition, renal deposition of IgG and IgM was visible in the model group of mice compared with control mice. The IF of kidneys in the AOA and prednisone groups was significantly weaker, indicating fewer glomerular deposits of IgG and IgM (Figures 5(b) and 5(c)). The fluorescence intensity analysis was performed to quantify the IgG and IgM deposition (Figures 5(e) and 5(f)). These results demonstrated that AOA-treated group mice suffered from slighter kidney damage than model group mice.

## 4. Discussion

In this study, we first demonstrated that AOA can inhibit ROR*γ*t transcriptional activity and the differentiation of Th17 cells. Furthermore, AOA demonstrated potent therapeutic effects in a mouse model of LN. AOA treatment significantly reduced the levels of serum anti-dsDNA antibody, as well as pathological damage and renal accumulation of antibodycomplex. Urine samples were not collected due to technical problems and the proteinuria was not evaluated in our study. Overall, these data demonstrated the therapeutic potent of AOA for the treatment of Th17-mediated inflammatory diseases.

LN is a challenging autoimmune disease associated with severe organ damage. Recent advances in the treatment of LN include the development of new immunosuppressants, traditional Chinese medicines, glucocorticoids (GCs), and stem cell transplantation. GCs, such as prednisone, hydrocortisone, and cortisone, have been widely used in the clinical practice in the treatment of LN patients. However, GCs have severe side effects leading to organ damage such as osteoporosis, infection, and cardiovascular disease [26]. Immunosuppressants, such as cyclophosphamide, methotrexate, cyclosporine, and leflunomide, have been used in combination with GCs to achieve good therapeutic results. However, they have a narrow therapeutic index and potentially serious toxicities, including bladder toxicity and infection [27]. Belimumab and rituximab have also been used in the clinical practice, but are plagued by concerns of potentially serious toxicities [28]. Hematopoietic stem cell transplantation is a novel investigational treatment strategy in its infancy. Therefore, further studies are required to develop novel, effective, and safe treatment options for patients with SLE.

Th17 cells are a subset of CD4^+^ T helper cells that produce IL-17A, IL-17F, IL-23, and other proinflammatory cytokines. Th17 cells have been shown to play a critical role in the pathogenesis of SLE. Compared with the wild-type mice, the BXD2 mice carry a higher percentage of Th17 cells, but not Th1 or Th2 cells in the spleen [29]. IL-17 overexpression enhanced disease, and IL-17R blockade can reduce its intensity in BXD2 mice [30]. Ets-1-/- mouse represents another model with lupus-like features, which demonstrated enhanced Th17 differentiation following Ets-1 deficiency [31, 32]. However, Schmidt et al. showed that IL-17A deficiency had no effect on the clinical course of lupus-prone MRL/lpr mice and NZB/NZW mice, but anti-IFN-*γ* treatment attenuated the severity of the LN [33]. We demonstrated that Nrf2 deficiency could promote Th17 differentiation and LN development in MRL/lpr mice with a C57BL/6 background, while another study showed contradictory results in the mix background mice [25]. We think the conflicting result from these studies may be due to the difference of genetic background in different models.

Many studies have shown that patients with SLE have elevated amounts of IL-17 in serum and plasma, with an increased frequency of Th17 cells in peripheral blood [34, 35]. Plasma IL-17 levels show a positive correlation with SLE disease activity [35]. Shah et al. showed that patients with SLE carried an increased portion of Th17 cells, whereas Th1 cells showed no variation [36]. Other studies also have shown that Th17 was closely related to lupus nephritis [37]. These evidences indicated that Th17 cells played an important role in LN, and IL-17 blocking may offer a therapeutic target for SLE.

ROR*γ*t is the master regulator of Th17 cell differentiation and the therapeutic target for autoimmune disorders. Hundreds of compounds have shown effective suppression of Th17 cell differentiation and function through directly inhibiting ROR*γ*t activity [38]. However, application of these compounds still stays in the preclinical stage; only a few clinical trials come into stage 1 or 2 due to the specificity crosstalk among ROR*γ*t and other nuclear receptors. Discovering more potent and specific ROR*γ*t antagonists still is underway. In this study, we found that AOA also suppressed ROR*γ*t transcriptional activity and Th17 cell differentiation and delayed LN clinical manifestation development, which demonstrated the druggable potent for autoimmune disease therapy. In future study may be needed to compare its therapeutic specificity with that of other inhibitors.

Treg cells evibit a potent immunosuppressive function and contribute to immunological tolerance against self-antigens by reducing the production of inflammatory cytokines. The deficiency of Treg cells can lead to the development of autoimmune diseases [39]. In this study, we also evaluated the expression of Foxp3, the critical transcription factor of Treg cells, and found that AOA only slightly affected Foxp3 expression (Figure S2). These results indicated that ameliorating Th17-mediated pathogenesis, rather than regulating Treg function, was the major regulation of AOA improving lupus nephritis clinical manifestation.

A significant number of therapeutic agents have been discovered from traditional Chinese medicine and natural products. Chinese scientists have reported the therapeutic effects of traditional artemisinin antimalarias, including artemisinin, dihydroartemisinin, artesunate, and artemether, in animal models of lupus as well as in patients [40, 41]. However, it has been reported that the overall efficacy of artemisinin in autoimmune diseases is weak and uncertain. Additionally, their insolubility also affects the absorption and bioavailability of oral administration. These challenges restrict the clinical application of artemisinin as a therapeutic drug for chronic autoimmune diseases. Another study already reported that ursolic acid (UA) can inhibit ROR*γ*t activity and prevent EAE development [17]. The structural backbone of AOA is similar to that of UA, we suggesting similar mechanisms of action in autoimmunologic disease therapy, which requires further investigation.

In conclusion, AOA is an effective inhibitor of ROR*γ*t, with potent inhibition on Th17 cell differentiation and secretion of IL-17A. Treatment with AOA ameliorated LN clinical manifestation in a pristane-induced mouse model, which suggested the potential therapeutic application of AOA in Th17-mediated inflammatory disease drug discovery.

## Data Availability

The data used to support the findings of this study are included within the article.

## Supplementary Materials

Supplementary Materials Figure S1: characterization of AOA. 3*β*-Acetyloxy-oleanoic acid (AOA) 1H NMR (400 MHz, CDCl3) *δ*5.24 (t, *J* = 3.1 Hz, 1H, H-12), 4.52-4.41 (m, 1H, H-3), 2.79 (dd, *J* = 13.7, 4.0 Hz, 1H, H-18), 2.02 (s, 3H, CH3COO), 1.95 (td, *J* = 13.3, 3.7 Hz, 1H), 1.89 -1.82 (m, 2H), 1.80-1.66 (m, 2H), 1.66 -1.47 (m, 9H), 1.47-1.13 (m, 7H), 1.10 (s, 3H,CH3-29), 1.04 (d, *J* = 13.6 Hz, 2H), 0.91 (s, 3H, CH3-27), 0.90 (s, 3H,CH3-23), 0.88 (s, 3H,CH3-30), 0.84 (s, 3H,CH3-26), 0.82 (s, 3H,CH3-25), 0.71 (s, 3H, CH3-24); 13C NMR (100 MHz, CDCl3) *δ* 184.53(-COOH), 171.28(OCOCH3), 143.81 (C-13), 122.75 (C-12), 81.13(C-3), 55.47(C-5), 47.74(C-18), 46.74(C-17), 46.02(C-9), 41.72(C-14), 41.07 (C-20), 39.46 (C-4), 38.24 (C-1), 37.89(C-8), 37.18 (C-10), 33.97(C-22), 33.27 (C-7), 32.69(C-19), 32.63(C-29), 30.87 (C-21), 28.24(C-23), 27.86 (C-2), 26.11(C- 15), 23.78(C-16), 23.71(C-27), 23.58(C-30), 23.04 (C-11), 21.53 (OCOCH3), 18.36(C-4), 17.38(C-24), 16.86(C-26), 15.58(C-25). Examination of the 1H NMR spectrum revealed seven methyl group signals at *δ*H 1.10 (s, 3H,CH3-29), 0.91 (s, 3H, CH3-27), 0.90 (s, 3H,CH3-23), 0.88 (s, 3H,CH3-30), 0.84 (s, 3H,CH3-26), 0.82 (s, 3H,CH3- 25), and 0.71 (s, 3H, CH3-24), which suggested that 1 belongs to a oleane-type triterpenoid. Resonances of the singlet methyl at *δ*H 2.02, together with carbonyl carbons at *δ*c 171.28, revealed acetate groups. The carbon signal at *δ*C 184.53 is assigned to a carboxyl group (-COOH). NMR signals at *δ*H5.24 (t, *J* = 3.1 Hz, 1H), *δ*C143.81, and 122.75 belong to a double bond. Figure S2: the effects of AOA on Th1, TH2, and Treg cells. (a-e) Naive CD4^+^CD25^−^ T cells were activated with anti-CD3 and CD28 under Th17 cell-polarizing conditions in the presence of AOA (0.625, 2.5, and 10 *μ*M) or DMSO. Five days later, cells were collected to test the mRNA levels of the transcription factors and cytokines of IFN-*γ*, Tbx21, IL-4, IL-13, and Foxp3. The mRNA expression was quantified and normalized to GAPDH. The quantitative real-time PCR were repeated 3 times with consistent results. The results are shown as mean ± SEM; ∗*p* < 0.05 and ∗∗*p* < 0.01. Table S1: the sequences of primer pairs for real-time RT-PCR. GAPDH: glyceraldehyde-3-phosphate dehydrogenase; IL: interleukin; IFN-*γ*: interferon gamma. The relative mRNA expression was detected by real-time PCR normalized to mouse GAPDH. All primers were synthesized by Shanghai Generay Biotech Co. Ltd. The methods used in this study are described in Materials and Methods.
