# Supplementary material for: 3β-Acetyloxy-oleanolic Acid Attenuates Pristane-Induced Lupus Nephritis by Regulating Th17 Differentiation
Source: J Immunol Res. 2019 May 22;2019:2431617. doi: 10.1155/2019/2431617 (PMC6556267; doi:10.1155/2019/2431617)
Supplement: Supplementary Materials — Figure S1: characterization of AOA. 3β-Acetyloxy-oleanoic acid (AOA) 1H NMR (400 MHz, CDCl3) δ5.24 (t, J = 3.1 Hz, 1H, H-12), 4.52-4.41 (m, 1H, H-3), 2.79 (dd, J = 13.7, 4.0 Hz, 1H, H-18), 2.02 (s, 3H, CH3COO), 1.95 (td, J = 13.3, 3.7 Hz, 1H), 1.89 -1.82 (m, 2H), 1.80-1.66 (m, 2H), 1.66 -1.47 (m, 9H), 1.47-1.13 (m, 7H), 1.10 (s, 3H,CH3-29), 1.04 (d, J = 13.6 Hz, 2H), 0.91 (s, 3H, CH3-27), 0.90 (s, 3H,CH3-23), 0.88 (s, 3H,CH3-30), 0.84 (s, 3H,CH3-26), 0.82 (s, 3H,CH3-25), 0.71 (s, 3H, CH3-24); 13C NMR (100 MHz, CDCl3) δ 184.53(-COOH), 171.28(OCOCH3), 143.81 (C-13), 122.75 (C-12), 81.13(C-3), 55.47(C-5), 47.74(C-18), 46.74(C-17), 46.02(C-9), 41.72(C-14), 41.07 (C-20), 39.46 (C-4), 38.24 (C-1), 37.89(C-8), 37.18 (C-10), 33.97(C-22), 33.27 (C-7), 32.69(C-19), 32.63(C-29), 30.87 (C-21), 28.24(C-23), 27.86 (C-2), 26.11(C- 15), 23.78(C-16), 23.71(C-27), 23.58(C-30), 23.04 (C-11), 21.53 (OCOCH3), 18.36(C-4), 17.38(C-24), 16.86(C-26), 15.58(C-25). Examination of the 1H NMR spectrum revealed seven methyl group signals at δH 1.10 (s, 3H,CH3-29), 0.91 (s, 3H, CH3-27), 0.90 (s, 3H,CH3-23), 0.88 (s, 3H,CH3-30), 0.84 (s, 3H,CH3-26), 0.82 (s, 3H,CH3- 25), and 0.71 (s, 3H, CH3-24), which suggested that 1 belongs to a oleane-type triterpenoid. Resonances of the singlet methyl at δH 2.02, together with carbonyl carbons at δc 171.28, revealed acetate groups. The carbon signal at δC 184.53 is assigned to a carboxyl group (-COOH). NMR signals at δH5.24 (t, J = 3.1 Hz, 1H), δC143.81, and 122.75 belong to a double bond. Figure S2: the effects of AOA on Th1, TH2, and Treg cells. (a-e) Naive CD4+CD25− T cells were activated with anti-CD3 and CD28 under Th17 cell-polarizing conditions in the presence of AOA (0.625, 2.5, and 10 μM) or DMSO. Five days later, cells were collected to test the mRNA levels of the transcription factors and cytokines of IFN-γ, Tbx21, IL-4, IL-13, and Foxp3. The mRNA expression was quantified and normalized to GAPDH. The quantitative real-time PCR were repeated 3 time [file 2431617.f1.docx]

**Supplementary information for：**

**3β-acetyloxy-oleanolic acid attenuates pristane-induced lupus nephritis by regulating Th17 differentiation**

Xiaoqing zhou^a, b, 1^, Huanpeng Chen^a, b, 1，^, Fengjiao Wei^a, b^, Qingyu Zhao^c^, Qiao Su^d^, Jinhao Liang^e^, Meng Yin^c^, Xuyan Tian^a, b^, Zhonghua Liu^f^, Bolan Yu^g^, Chuan Bai^a^, Xixin He^e, *^and Zhaofeng Huang^a, b, *^

^a^ Institute of Human Virology, Sun Yat-sen University, Guangzhou, China

^b^ Department of Biochemistry and molecular biology, Zhongshan School of Medicine, Sun Yat-sen University, Guangzhou, China

^c^ Sun Yat-sen University Cancer Center, Guangzhou, China

^d^ Animal Experiment Center, The First Affiliated Hospital of Sun Yat-sen University, Guangzhou, China

^e^ School of Pharmaceutical Sciences, Guangzhou University of Chinese Medicine, Guangzhou, China

^f^ Animal Experiment Center, South China Agricultural University, Guangzhou, China

^g^ Key Laboratory for Major Obstetric Diseases of Guangdong Province, Third Affiliated Hospital of Guangzhou Medical University, Guangzhou, China.

^1^ there authors contribute equally to this study.

**Corresponding authors**:

Dr. Zhaofeng Huang, address is: N1311 Rm, No.10 Bld, 74 Zhongshan 2nd Rd, Guangzhou, 510080, China, Tel: 8620-87335529, Fax: 8620-87332588, E-mail: [hzhaof@mail.sysu.edu.cn](mailto:hzhaof@mail.sysu.edu.cn)

Dr. Xixin He, address is+: Room C118, Pharmaceutical building, School of Pharmaceutical Sciences, Guangzhou University of Chinese Medicine, 232#, Waihuan Dong Road, High Education Mega Center, Guangzhou 510006

P. R. China Tel: +86-20-3935-8073 E-mail: [mark07@gzucm.edu.cn](mailto:mark07@gzucm.edu.cn)

**Supplementary Figure1.**


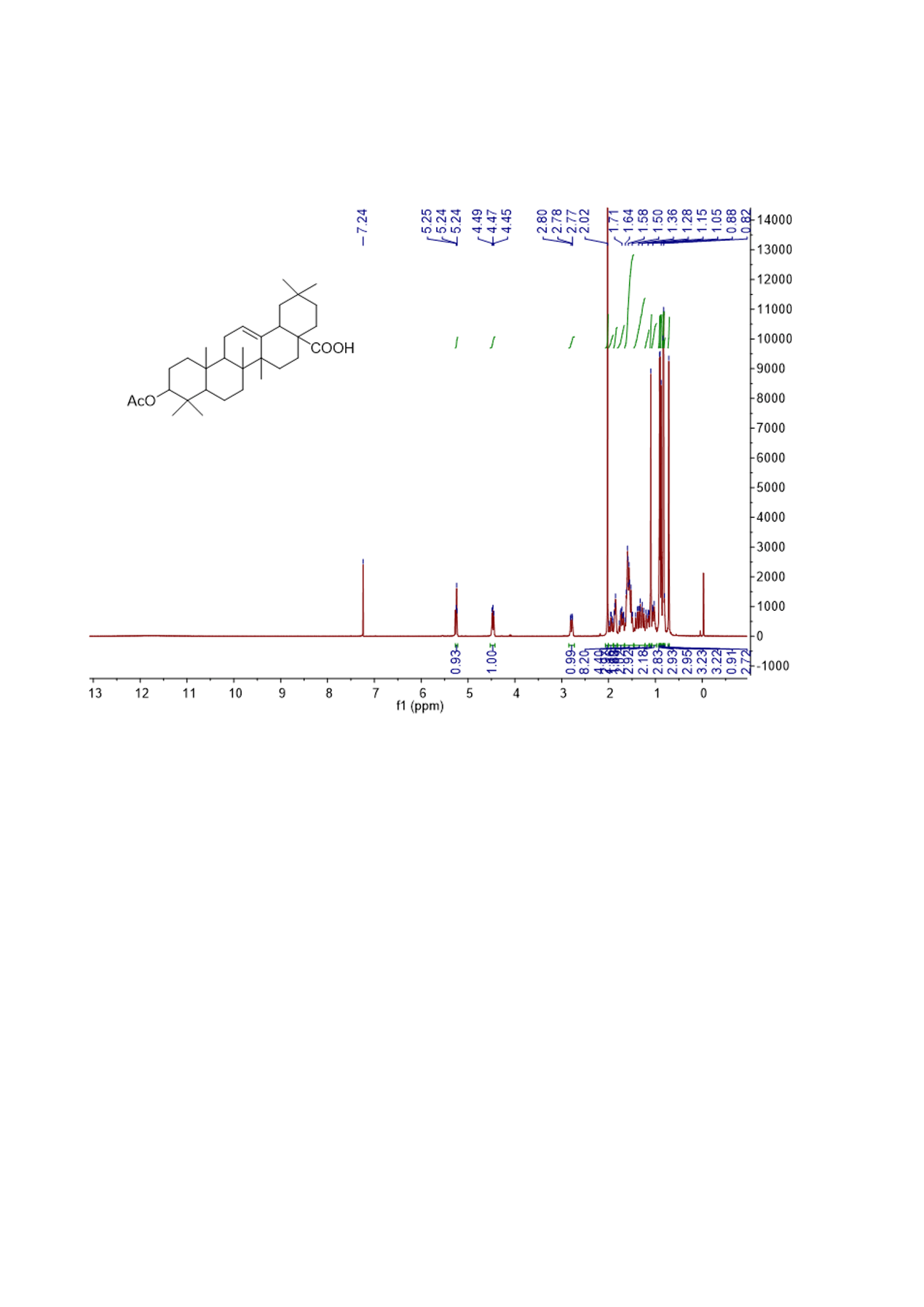


^1^H-NMR spectrum of AOA (400MHz, CDCl_3_)


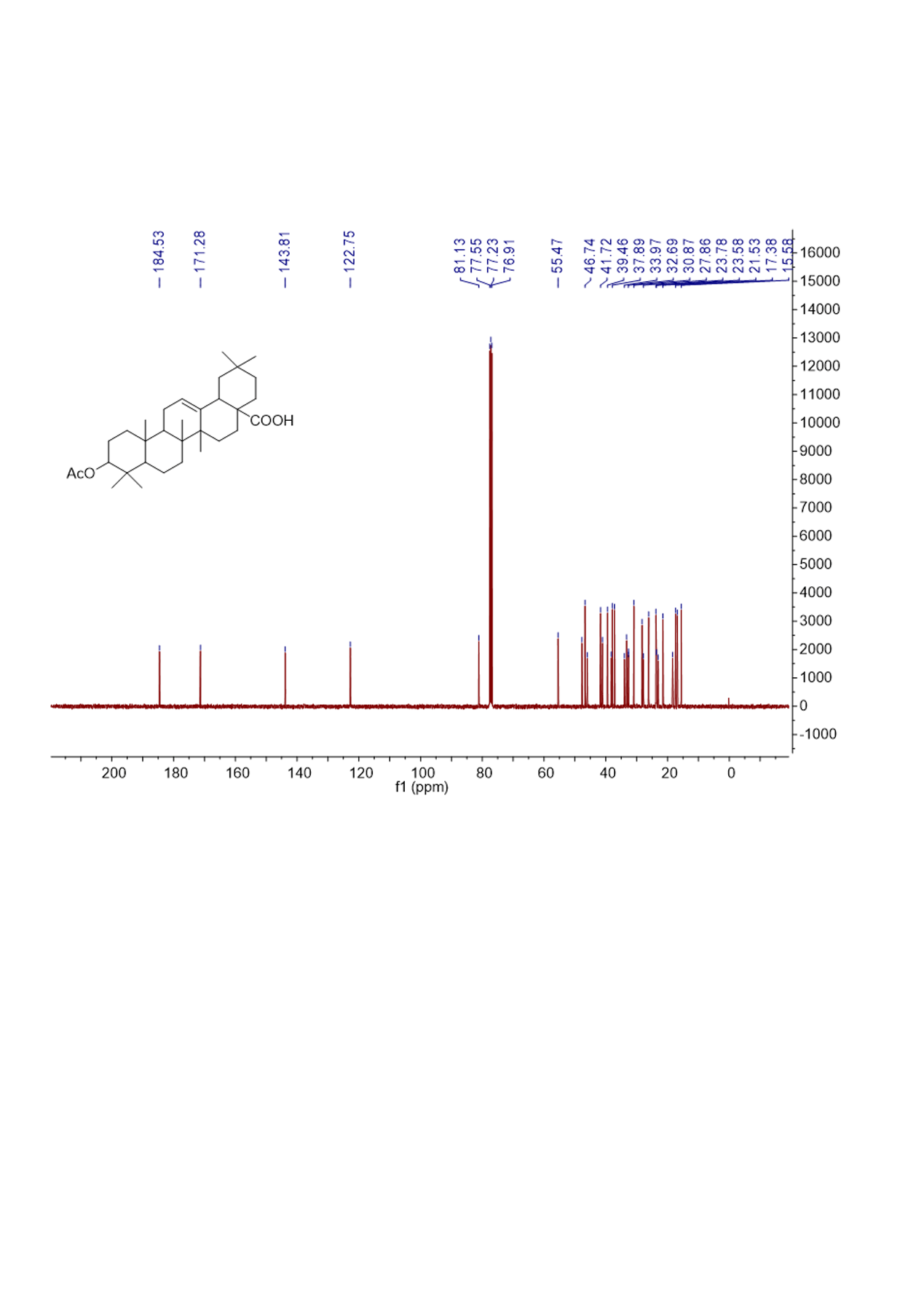


^13^C-NMR spectrum of AOA (100MHz, CDCl_3_)

**Supplementary Figure1.Characterization of AOA**.

**3β-acetyloxy-oleanoic acid (AOA)** ^1^H NMR (400 MHz, CDCl_3_) δ5.24 (t, *J* = 3.1 Hz, 1H, H-12), 4.52-4.41 (m, 1H, H-3), 2.79 (dd, *J* = 13.7, 4.0 Hz, 1H, H-18), 2.02 (s, 3H, CH_3_COO), 1.95 (td, *J* = 13.3, 3.7 Hz, 1H), 1.89 -1.82 (m, 2H), 1.80-1.66 (m, 2H), 1.66 -1.47 (m, 9H), 1.47-1.13 (m, 7H), 1.10 (s, 3H,CH_3_-29), 1.04 (d, *J* = 13.6 Hz, 2H), 0.91 (s, 3H, CH_3_-27), 0.90 (s, 3H,CH_3_-23), 0.88 (s, 3H,CH_3_-30), 0.84 (s, 3H,CH_3_-26), 0.82 (s, 3H,CH_3_-25), 0.71 (s, 3H, CH_3_-24); ^13^C NMR (100 MHz, CDCl_3_) δ 184.53(-COOH), 171.28(OCOCH_3_), 143.81 (C-13), 122.75 (C-12), 81.13(C-3), 55.47(C-5), 47.74(C-18), 46.74(C-17), 46.02(C-9), 41.72(C-14), 41.07 (C-20), 39.46 (C-4), 38.24 (C-1), 37.89(C-8), 37.18 (C-10), 33.97(C-22), 33.27 (C-7), 32.69(C-19), 32.63(C-29), 30.87 (C-21), 28.24(C-23), 27.86 (C-2), 26.11(C-15), 23.78(C-16), 23.71(C-27), 23.58(C-30), 23.04 (C-11), 21.53 (OCOCH_3_), 18.36(C-4), 17.38(C-24), 16.86(C-26), 15.58(C-25).

Examination of the ^1^H NMR spectrum revealed seven methyl groups signals at *δ*_H_ 1.10 (s, 3H,CH_3_-29), 0.91 (s, 3H, CH_3_-27), 0.90 (s, 3H,CH_3_-23), 0.88 (s, 3H,CH_3_-30), 0.84 (s, 3H,CH_3_-26), 0.82 (s, 3H,CH_3_-25), 0.71 (s, 3H, CH_3_-24), which suggested that 1 belongs to a oleane-type triterpenoid. Resonances of the singlet methyl at *δ*_H_ 2.02, together with a carbonyl carbons at *δ*_c_ 171.28, revealed an acetate groups. The carbon signal at δ_C_ 184.53 is assigned to a [carboxyl](https://fanyi.so.com/?src=onebox#carboxyl) group (-COOH). NMR signals at δ_H_5.24 (t, *J* = 3.1 Hz, 1H), δ_C_143.81 and 122.75 belong to a double bond.

**Supplementary Figure2.**


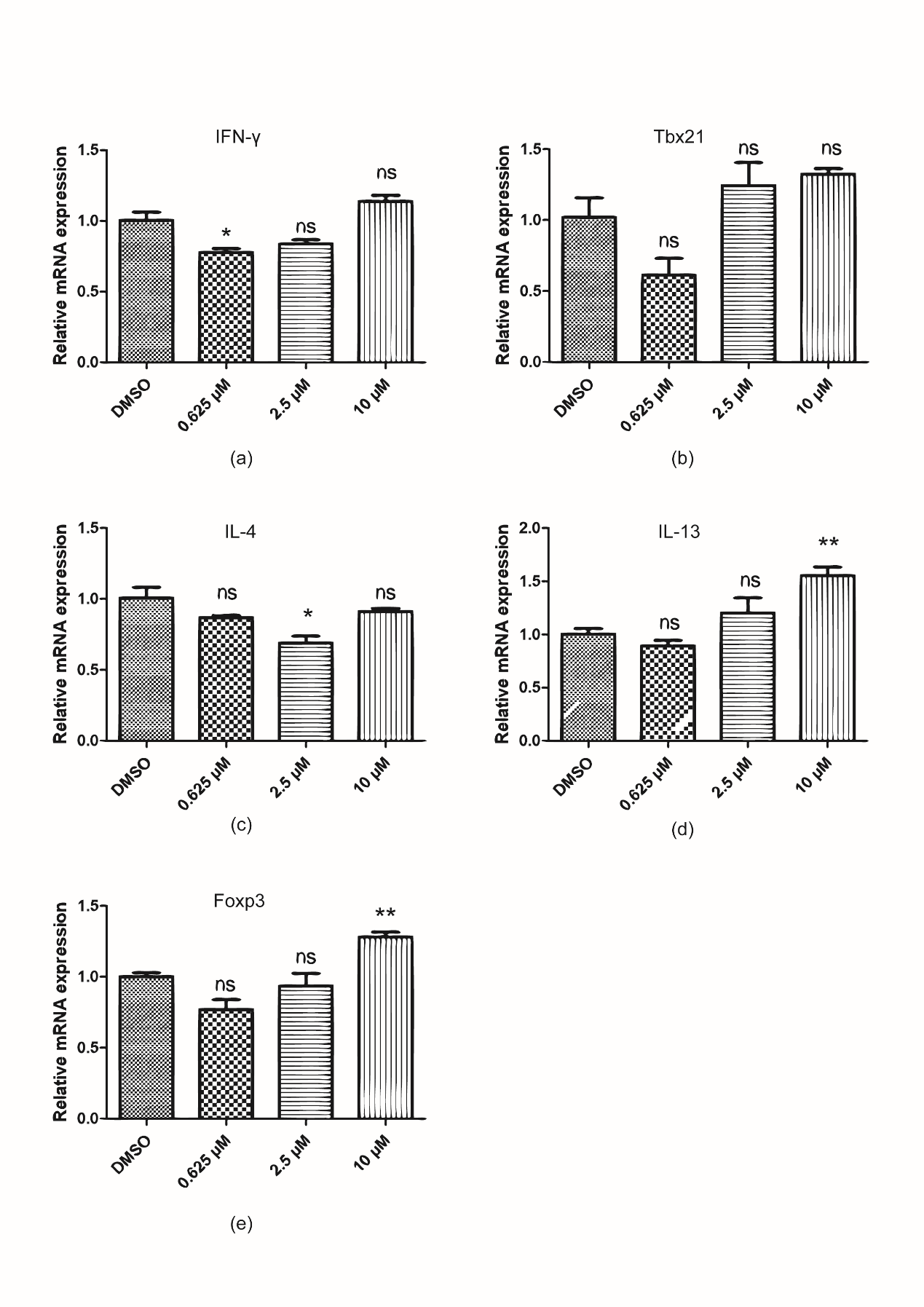


**Figure S2. The Effects of AOA on Th1, TH2 and Treg cells**

(a-e) Naive CD4+CD25-T cells were activated with anti-CD3 and CD28 under Th17 cell-polarizing conditions in the presence of AOA (0.625, 2.5, 10 μM) or DMSO. 5 days later, cells were collected to test the mRNA levels of the transcription factors and cytokines of IFN-γ, Tbx21, IL-4, IL-13, Foxp3. The mRNA expression was quantified and normalized to GAPDH. The quantitative real-time PCR were repeated 3 times with consistent results. The results are shown as mean ± SEM; * *p* < 0.05; ** *p* < 0.01.

**Supplementary Table 1**

**Table S1.** The sequences of primer pairs for Real-Time RT-PCR

| Target gene | Forward sequence | Reverse sequence |
| --- | --- | --- |
| RORγt | 5’-AAACTTGACAGCATCTCGGGA-3’ | 5’-TGCAGGAGTAGGCCACATTACA-3’ |
| IL-17A | 5’-CTCCAGAAGGCCCTCAGACTAC -3’ | 5’-AGCTTTCCCTCCGCATTGACACAG -3’ |
| IL-17F | 5’-GAGGATAACACTGTGAGAGTTGAC-3’ | 5’-GAGTTCATGGTGCTGTCTTCC-3’ |
| IL-22 | 5’-ATGAGTTTTTCCCTTATGGGGAC-3’ | 5’-GCTGGAAGTTGGACACCTCAA-3’ |
| IFN-γ | 5’- GCCACGGCACAGTCATTGA -3’ | 5’- TGCTGATGGCCTGATTGTCTT -3’ |
| Foxp3 | 5’-GGCCCTTCTCCAGGACAGA-3’ | 5’-GCTGATCATGGCTGGGTTGT-3’ |
| IL-4 | 5’- GGTCTCAACCCCCAGCTAGT -3’ | 5’- GCCGATGATCTCTCTCAAGTGAT -3’ |
| IL-13 | 5’-CCTGGCTCTTGCTTGCCTT-3’ | 5’-GGTCTTGTGTGATGTTGCTCA-3’ |
| Tbx21 | 5’-AGCAAGGACGGCGAATGTT-3’ | 5’-AGCAAGGACGGCGAATGTT-3’ |
| GAPDH | 5’-TGGTGAAGGTCGGTGTGAAC-3’ | 5’-GGGTGGACATATAAGCGGTTC-3’ |

**Table S1.** The sequences of primer pairs for Real-Time RT-PCR. GAPDH: glyceraldehyde-3-phosphate dehydrogenase; IL: Interleukin; IFN-γ: interferon gamma. The relative mRNA expression was detected by Real-Time PCR with normalizing to mouse GAPDH. All primers were synthesized by Shanghai Generay Biotech Co, Ltd. The methods used in this study are described in materials and methods.
